# Prevalence of common aneuploidy in twin pregnancies

**DOI:** 10.1038/s10038-021-01001-0

**Published:** 2022-01-01

**Authors:** Akiko Konishi, Osamu Samura, Jin Muromoto, Yoko Okamoto, Hironori Takahashi, Yasuyo Kasai, Mayuko Ichikawa, Naoki Yamada, Noriko Kato, Hiroshi Sato, Hiromi Hamada, Naoyuki Nakanami, Maya Machi, Kiyotake Ichizuka, Rei Sunami, Toshitaka Tanaka, Naoto Yonetani, Yoshimasa Kamei, Takeshi Nagamatsu, Mariko Matsumoto, Shinya Tairaku, Arisa Fujiwara, Hiroaki Nakamura, Takashi Harada, Takafumi Watanabe, Shoko Sasaki, Satoshi Kawaguchi, Sawako Minami, Masaki Ogawa, Kiyonori Miura, Nobuhiro Suzumori, Junya Kojima, Tomomi Kotani, Rumi Sasaki, Tsukasa Baba, Aya Toyofuku, Masayuki Endo, Naoki Takeshita, Takeshi Taketani, Masakatsu Sase, Keiichi Matsubara, Kei Hayata, Yoshinobu Hamada, Makiko Egawa, Toshiyuki Kakinuma, Sachio Matsushima, Michihiro Kitagawa, Tomomi Shiga, Ryuhei Kurashina, Hironori Hamada, Hiroaki Takagi, Akane Kondo, Norio Miharu, Michiko Yamashita, Madoka Horiya, Keiji Morimoto, Ken Takahashi, Aikou Okamoto, Akihiko Sekizawa, Haruhiko Sago

**Affiliations:** 1 Department of Obstetrics and Gynecology, The Jikei University School of Medicine, Tokyo, Japan; 2 Center for Maternal-Fetal, Neonatal and Reproductive Medicine, National Center for Child Health and Development, Tokyo, Japan; 3 Department of Maternal Fetal Medicine, Osaka Women’s and Children’s Hospital, Osaka, Japan; 4 Department of Obstetrics and Gynecology, Jichi Medical University Hospital, Shimotsuke, Japan; 5 Department of Obstetrics and Gynecology, Japanese Red Cross Medical Center, Tokyo, Japan; 6 Department of Obstetrics and Gynecology, Nara Medical University Hospital, Kashihara, Japan; 7 Department of Obstetrics and Gynecology, Mito Saiseikai General Hospital, Mito, Japan; 8 Department of Obstetrics and Gynecology, Japanese Red Cross Aichi Medical Center Nagoya Daini Hospital, Nagoya, Japan; 9 Department of Obstetrics and Gynecology, Hyogo Prefectural Amagasaki General Medical Center, Amagasaki, Japan; 10 Department of Obstetrics and Gynecology, University of Tsukuba Hospital, Tsukuba, Japan; 11 Department of Obstetrics, Fukuoka Children’s Hospital, Fukuoka, Japan; 12 Department of Obstetrics and Gynecology, Showa University Hospital, Tokyo, Japan; 13 Department of Obstetrics and Gynecology, Showa University Northern Yokohama Hospital, Yokohama, Japan; 14 Department of Obstetrics and Gynecology, Yamanashi Prefectural Central Hospital, Kofu, Japan; 15 Department of Obstetrics and Gynecology, Juntendo University Shizuoka Hospital, Izunokuni, Japan; 16 Department of Obstetrics and Gynecology, Tokushima University Hospital, Tokushima, Japan; 17 Department of Obstetrics and Gynecology, Saitama Medical University, Saitama, Japan; 18 Department of Obstetrics and Gynecology, The University of Tokyo Hospital, Tokyo, Japan; 19 Department of Obstetrics and Gynecology, Japanese Red Cross Kyoto Daiichi Hospital, Kyoto, Japan; 20 Department of Obstetrics, Hyogo Prefectural Kobe Children’s Hospital, Kobe, Japan; 21 Department of Obstetrics and Gynecology, National Hospital Organization Kyushu Medical Center, Fukuoka, Japan; 22 Department of Obstetrics and Gynecology, Osaka City General Hospital, Osaka, Japan; 23 Department of Obstetrics and Gynecology, Tottori University Hospital, Yonago, Japan; 24 Department of Obstetrics and Gynecology, Fukushima Medical University, Fukushima, Japan; 25 Department of Obstetrics and Gynecology, Mitsubishi Kyoto Hospital, Kyoto, Japan; 26 Department of Obstetrics and Gynecology, Hokkaido University Hospital, Sapporo, Japan; 27 Department of Obstetrics and Gynecology, Wakayama Medical University Hospital, Wakayama, Japan; 28 Department of Obstetrics and Gynecology, Tokyo Women’s Medical University Hospital, Tokyo, Japan; 29 Department of Obstetrics & Gynecology, Nagasaki University Graduate School of Biomedical Sciences, Nagasaki, Japan; 30 Department of Obstetrics and Gynecology, Nagoya City University Graduate School of Medical Sciences, Nagoya, Japan; 31 Department of Obstetrics and Gynecology, Tokyo Medical University Hospital, Tokyo, Japan; 32 Center for Maternal-Neonatal Care, Nagoya University Hospital, Nagoya, Japan; 33 Department of Obstetrics and Gynecology, Faculty of Life Sciences, Kumamoto University, Kumamoto, Japan; 34 Department of Obstetrics and Gynecology, Iwate Medical University Hospital, Iwate, Japan; 35 Department of Obstetrics and Gynecology, Japanese Red Cross Wakayama Medical Center, Wakayama, Japan; 36 Department of Obstetrics and Gynecology, Osaka University Hospital, Suita, Japan; 37 Department of Obstetrics and Gynecology, Toho University Sakura Medical Center, Sakura, Japan; 38 Department of Pediatrics, Shimane University Hospital, Izumo, Japan; 39 Department of Obstetrics and Gynecology, Yamaguchi Prefectural Grand Medical Center, Hofu, Japan; 40 Department of Regional Pediatrics and Perinatology, Ehime University Graduate School of Medicine, Toon, Japan; 41 Department of Obstetrics and Gynecology, Okayama University Hospital, Okayama, Japan; 42 Department of Obstetrics and Gynecology, Dokkyo Medical University Saitama Medical Center, Koshigaya, Japan; 43 Department of Nutrition and Metabolism in Cardiovascular Disease, Graduate School of Medical and Dental Sciences, Tokyo Medical and Dental University, Tokyo, Japan; 44 Department of Obstetrics and Gynecology, International University of Health and Welfare Hospital, Nasushiobara, Japan; 45 Department of Obstetrics and Gynecology, Kochi Medical School Hospital, Nankoku, Japan; 46 Sanno Birth Center, Tokyo, Japan; 47 Department of Obstetrics and Gynecology, Gifu University Hospital, Gifu, Japan; 48 Department of Obstetrics and Gynecology, Nippon Medical School Hospital, Tokyo, Japan; 49 Adachi Hospital, Kyoto, Japan; 50 Department of Obstetrics and Gynecology, Kanazawa Medical University Hospital, Kahoku, Ishikawa Japan; 51 Perinatal Medical Center, Shikoku Medical Center for Children and Adults, National Hospital Organization, Zentsuji, Kagawa Japan; 52 Department of Obstetrics and Gynecology, Chuden Hospital, Hiroshima, Japan; 53 Department of Obstetrics and Gynecology, Kansai Rosai Hospital, Amagasaki, Japan; 54 Department of Obstetrics and Gynecology, The Jikei University Kashiwa Hospital, Kashiwa, Japan; 55 Department of Obstetrics and Gynecology, The Jikei University Daisan Hospital, Tokyo, Japan

**Keywords:** Chromosome abnormality, Genetics research

## Abstract

The incidence of chromosomal abnormalities in twin pregnancies is not well-studied. In this retrospective study, we investigated the frequency of chromosomal abnormalities in twin pregnancies and compared the incidence of chromosomal abnormalities in dichorionic diamniotic (DD) and monochorionic diamniotic (MD) twins. We used data from 57 clinical facilities across Japan. Twin pregnancies of more than 12 weeks of gestation managed between January 2016 and December 2018 were included in the study. A total of 2899 and 1908 cases of DD and MD twins, respectively, were reported, and the incidence of chromosomal abnormalities in one or both fetuses was 0.9% (25/2899) and 0.2% (4/1908) in each group (*p* = 0.004). In this study, the most common chromosomal abnormality was trisomy 21 (51.7% [15/29]), followed by trisomy 18 (13.8% [4/29]) and trisomy 13 (6.9% [2/29]). The incidence of trisomy 21 in MD twins was lower than that in DD twins (0.05% vs. 0.5%, *p* = 0.007). Trisomy 21 was less common in MD twins, even when compared with the expected incidence in singletons (0.05% vs. 0.3%, RR 0.15 [95% CI 0.04–0.68]). The risk of chromosomal abnormality decreases in twin pregnancies, especially in MD twins.

## Introduction

The incidence of twin pregnancies, particularly dizygotic pregnancies, which positively correlate with the maternal age and use of assisted reproductive technologies (ART), has increased [1]. Twin pregnancies have a higher risk of intrauterine growth retardation, preterm delivery, and neonatal mortality. Many studies have found that twin pregnancies also have a higher risk of congenital anomalies than singleton pregnancies [2–4]. Approximately 25% of congenital anomalies are attributed to chromosomal abnormalities [5], and twin pregnancies have also been considered to have a high risk of chromosomal abnormalities. However, it was reported that the incidence of trisomy 21 was lower in twin than in singleton pregnancies, most notably in monozygotic twins [6], although these findings were not universal. The risk of chromosomal abnormalities in twin pregnancies remains controversial.

This study retrospectively investigated the frequency of chromosomal abnormalities in twin pregnancies in Japan. The incidence of chromosomal abnormalities in dichorionic diamniotic (DD) and monochorionic diamniotic (MD) twins were compared using data collected from 57 clinical facilities across Japan. We also compared the observed incidence of trisomy 21 among twin pregnancies with that expected based on maternal age-matched singletons.

## Materials and methods

This retrospective observational study used data collected from 57 perinatal centers that handle twin deliveries all over Japan. We asked facilities that handle many deliveries in Japan to participate in this research, and 57 of them cooperated. Cases of twin pregnancies between January 2016 and December 2018, wherein chorionicity was determined using ultrasound examination and that continued beyond 12 weeks of gestation, were included in the study. Cases of spontaneous abortion, artificial stillbirth, and single fetal demise after 12 weeks of gestation were not excluded. Cases of unknown age at the time of ovum collection and cases of ovum donation were excluded because the risk of chromosomal abnormalities in these cases may not be correlated with maternal age. Cases of unknown chorionicity, monochorionic monoamniotic (MM) twins, vanishing twins, and multifetal pregnancy reduction were also excluded. This study was approved by the ethics institutional boards of the Jikei University, School of Medicine, Tokyo, Japan (approval number: 31-175 [9674]). Disclosure of this study was opt-out at each institution.

We collected the data for the number of total and twin deliveries in addition to that of the twins, including maternal age, method of conception, karyotype, and pregnancy outcomes from each facility. Chromosomal abnormalities were diagnosed by karyotyping using chorionic villus or amniotic fluid, prenatally, or peripheral blood, postnatally. Most of these tests were offered in case of abnormal clinical findings, including prenatal ultrasound findings and postnatal dysmorphic features. These tests were also sought by women of advanced maternal age (AMA; ≥ 35 years), even in the absence of abnormal findings. In cases of positive noninvasive prenatal testing (NIPT) results for women of AMA further investigations such as amniocentesis was performed. If there were no findings suggestive of chromosomal abnormalities during the fetal or neonatal period, chromosomal tests were not performed and the case was deemed to have “no chromosomal abnormalities”. Karyotype annotation was in accordance with the International System for Human Cytogenomic Nomenclature 2016.

Statistical analysis was performed using the EZR software (Saitama Medical Center, Jichi Medical University, Shimotsuke, Japan) and statistical significance was set at *p* < 0.05. The expected incidence of trisomy 21 in twin pregnancies was calculated by extrapolating from maternal age-matched risk using the Morris model [7]. The Morris model calculates the risk of Down syndrome using the following formula: risk = 1/(1 + exp (7.330 − 4.211/(1 + exp (−0.282 × (age − 37.23))))). Relative risks (RR) with 95% confidence intervals (95% CI) were estimated to compare the incidence of trisomy 21 between twin pregnancies and singleton pregnancies. RR were adjusted for maternal age divided into 1-year blocks.

## Results

**Fig. 1:**
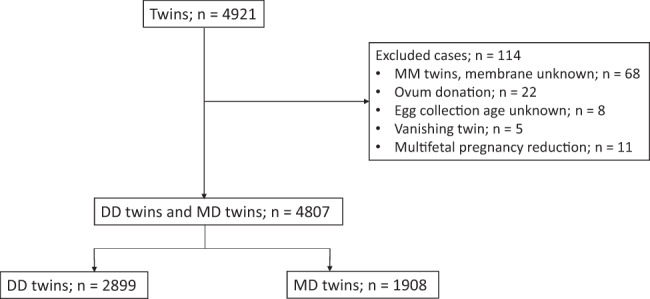
Number and types of twin pregnancies included in the study. The number and types of twin pregnancies included in the study, as well as the excluded cases and the reason for exclusion, are summarized. DD Dichorionic diamniotic, MD Monochorionic diamniotic, and MM Monochorionic monoamniotic.

The study cohort included 120,927 deliveries from 57 facilities across Japan, among which 4921 (4.1%) were twin pregnancies. A total of 114 cases were excluded from the analysis, of which 68 cases were of MM twins and of unknown chorionicity, 22 cases were of ovum donation, 8 cases were of unknown age at the time of ovum collection, 5 cases were of vanishing twins, and 11 cases were of reduced fetuses. Of the 4807 included twin pregnancies, 2899 (60.3%) were DD twins and 1908 (39.7%) were MD twins (Fig. 1).

**Fig. 2:**
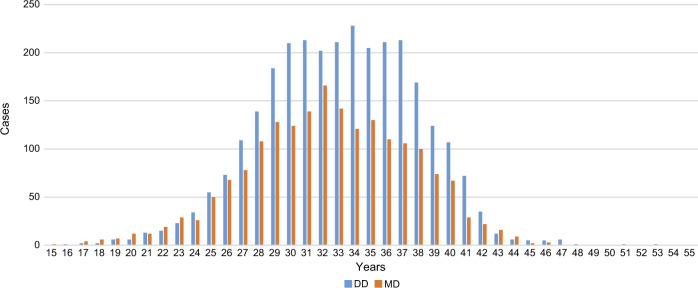
Maternal age distribution in different groups. The number of mothers of DD and MD twins and the maternal age is displayed. DD Dichorionic diamniotic and MD Monochorionic diamniotic

Table 1 shows a comparison of the maternal characteristics of DD and MD twins. The mean maternal age was 33.1 ± 4.8 and 32.4 ± 5.1 years in DD and MD twins, respectively (*p* < 0.001). As for the method of conception, MD twins had significantly more natural pregnancies (*p* < 0.001), whereas more than half of DD twins were conceived through infertility treatment. The maternal age distribution of DD and MD twins are shown in Fig. 2.

**Table 1:** Maternal characteristics of twin pregnancies.

	DD twins (*n* = 2899)	MD twins (*n* = 1908)	*p*-value
Maternal age (mean ± SD years)	33.1 ± 4.8	32.4 ± 5.1	<0.001
Conception mode (% of total pregnancies studied)			<0.001
Natural conception	1333 (46.0)	1473 (77.2)	
Infertility treatment	1566 (54.0)	435 (22.8)	
Ovulation drugs	495 (17.1)	66 (3.5)	
AIH	263 (9.1)	44 (2.3)	
IVF-ET	509 (17.6)	213 (11.2)	
ICSI	258 (8.9)	92 (5.1)	
Other	41 (1.4)	20 (0.9)	
Gestational age at delivery (mean ± SD weeks)	35.7 ± 3.0	34.6 ± 4.6	<0.001

Abbreviations: *AIH* Artificial insemination with donor semen, *DD* Dichorionic diamniotic,

*ICSI* Intracytoplasmic sperm injection, *IVF-ET* In vitro fertilization and embryo transfer,

*MD* Monochorionic diamniotic, and *SD* Standard deviation.

The incidence of chromosomal abnormalities in DD and MD twins is shown in Table 2. We classified chromosomal abnormalities into three types: recognizable phenotype, mild phenotype, and no phenotype. The incidence of chromosomal abnormalities in one or both fetuses were 0.9% (25/2899) in DD twins, which was significantly higher than that in MD twins (0.2% [4/1908] (*p* = 0.004). The most common chromosomal abnormality was trisomy 21 (51.7% [15/29]), followed by trisomy 18 (13.8% [4/29]), and trisomy 13 (6.9% [2/29]). The incidence of trisomy 21 in DD twins was significantly higher than that in MD twins (0.5% vs. 0.05%, *p* = 0.007). Trisomy 21 was identified in only one fetus of the twin pairs in DD twins; however, trisomy 21 occurred in both fetuses of one pair in MD twins. There were no significant differences in the frequency of chromosomal abnormalities other than trisomy 21 between DD and MD twins. Eleven cases (8 DD and 3 MD twins) had abnormal findings, such as cardiac malformations and multiple malformations; however, the results of karyotyping were normal.

**Table 2:** The incidence of chromosomal abnormalities.

Abnormal karyotype	Total *N* = 4807	DD twins *N* = 2899	MD twins *N* = 1908	*p*-value
Recognizable phenotype				
Trisomy 21	15 (0.3)	14 (0.5)	1* (0.05)	0.007
Trisomy 18	4 (0.08)	3 (0.1)	1 (0.05)	1.000
Trisomy 13	2 (0.04)	2 (0.07)	0	0.521
Additional material on the chromosome^a^	1 (0.02)	0	1 (0.05)	
Marker chromosome^b^	2 (0.04)	1 (0.03)	1 (0.05)	
Deletion^c^	1 (0.02)	1 (0.03)	0	
Mild phenotype				
Sex chromosomal aneuploidy	0	0	0	
Mosaic aneuploidy^d^	2 (0.04)	2 (0.07)	0	
No phenotype				
Inversion^e^	1 (0.02)	1 (0.03)	0	
Robertsonian translocation^f^	1 (0.02)	1* (0.03)	0	
Total	29 (0.6)	25 (0.9)	4 (0.2)	0.004

*Both fetuses were diagnosed with chromosomal abnormalities.

Abbreviations: *DD* Dichorionic diamniotic, *MD* Monochorionic diamniotic.

^a^46,XY,der(11)t(11;12)(q24.1;11.1).

^b^47,X, + 2mar[8]/46,X, + mar[6]/48,X, + 3mar[2]/48,XX, +2mar[1]. 47,XY, + mar.

^c^46,XY,del(18)(q23).

^d^47,XX, + 21[4]/46,XX[12]. 47,XX, + 4[17]/47,XX[33].

^e^46,XX,inv(1)(p22p35).

^f^45,XY,der(13;14)(q10:q10).

Table 3 shows a comparison of the observed and expected incidence of trisomy 21 per fetus in each group. The observed incidence of trisomy 21 was significantly lower than the expected incidence in both MD (0.05% vs. 0.3%, RR 0.15 [95% CI 0.04–0.68]) and total (0.2% vs. 0.4%, RR 0.45 [95% CI 0.25–0.83]) twins. However, there were no significant differences between the observed and expected incidence of trisomy 21 in DD twins (0.2% vs. 0.4%, RR 0.64 [95% CI 0.33–1.24]).

**Table 3:** Comparison of the incidences of trisomy 21 per fetus between observed and expected.

	Number of trisomy 21 fetuses		
	Observed incidence (%)	Expected incidence (%)*	RR observed versus expected (95% CI)
DD twins	14 (0.2)	21.7 (0.4)	0.64 (0.33-1.24)
MD twins	2 (0.05)	12.9 (0.3)	0.15 (0.04-0.68)
Total Twins	16 (0.2)	34.6 (0.4)	0.46 (0.25-0.83)

^*^Expected incidence of trisomy 21 calculated by maternal age-matched singleton rates using the Morris model (Morris, 2002).

Abbreviations: *DD* Dichorionic diamniotic and *MD* Monochorionic diamniotic.

## Discussion

This retrospective investigation provides an overview of the rates of chromosomal abnormalities in twin pregnancies after 12 weeks of gestation. In this study, approximately half of the chromosomal abnormalities were trisomy 21. We found that the incidence of chromosomal abnormalities in MD twins was lower than that in DD twins, and the incidence of trisomy 21 in MD twins was lower than that in DD twins. We also compared the observed incidence of trisomy 21 for twin pregnancies with the expected incidence based on maternal age-matched singletons and found that the observed incidence was lower than the expected incidence, especially in MD twins.

Half of the chromosomal abnormalities found in twin pregnancies in this study included trisomy 21 (51.7% [15/29]), followed by trisomy 18 (13.8% [4/29]), and trisomy 13 (6.9% [2/29]). These findings were similar to the general frequency in fetuses or neonates reported previously [8, 9]. No cases of sex chromosome aneuploidy were found in this study. It is suspected that neonates with sex chromosome aneuploidy were not karyotyped because they showed no dysmorphic features.

In this study, MD twins were found to have a significantly lower incidence of chromosomal abnormalities compared with DD twins (0.9% vs. 0.2%, *p* = 0.004). The incidence of trisomy 21 was also significantly lower in MD twins than in DD twins (0.5% vs. 0.05%, *p* = 0.007). Monozygotic twins have a reportedly lower frequency of trisomy 21 compared with dizygotic twins [6], although monozygotic twins are not exactly the same as MD twins. Of note, 25–30% of monozygotic twins are DD twins [10].

We found that the risk of trisomy 21 is lower in twins, especially in MD twins than in singleton pregnancies. The incidence of chromosomal abnormalities was found to be lower in twins than that reported in singleton pregnancies [8, 9], contrasting with previous reports that suggested that twins had a higher risk of chromosomal abnormalities [11, 12]. Some studies from America and Europe showed a lower risk of trisomy 21 in twins than in singletons. There was only one report which mentioned that the ratio of observed-to-expected trisomy 21 incidence per pregnancy for monozygotic, dizygotic, and all twins was 33.6%, 75.2%, and 70.0%, respectively [6]. In our study, the observed incidence of trisomy 21 was significantly lower than the expected incidence in both MD twins (0.05% vs. 0.3%, RR 0.15 [95% CI 0.04–0.68]) and total twins (0.2% vs. 0.4%, RR 0.45 [95% CI 0.25–0.83]).

The low frequency of chromosomal abnormalities in twin pregnancies could be attributed to the fact that many twin pregnancies are lost or converted to singleton pregnancies [13–15]. Most twins are lost very early in the pregnancy. One study estimated that only one in eight individuals originating as a twin actually goes on to be born as a twin [16]. Furthermore, the abortion rate in twin pregnancies is three times higher than in singleton pregnancies [17, 18]. In addition, in twin pregnancies, embryos and fetuses with chromosomal abnormalities are likely to be eliminated, resulting in singleton pregnancies or miscarriages [10]. Therefore, the incidence of chromosomal abnormalities in twin pregnancies that reach 12 weeks of gestation is likely to be lower than that in singleton pregnancies, and our results support this argument. Early pregnancy loss is significantly more common in monochorionic than in dichorionic twins and, in the setting of concordance for aneuploidy, an even higher risk of loss may have contributed to the relatively low incidence of Down syndrome in monozygotic pregnancies [19, 20].

This study has some limitations given the nature of the data used and the study design. Since this study included only cases where fetal heartbeats were confirmed after 12 weeks of gestation, cases of spontaneous abortion before the diagnosis of chromosomal abnormalities as well as of induced abortion without fetal chromosomal examination were excluded. In addition, some cases deemed to have “no chromosomal abnormalities” might have had chromosomal abnormalities with minor phenotypic manifestations, mosaicism, or sex chromosome abnormalities, since chromosomal tests were not performed in all cases. In cases without karyotyping, we diagnosed “no chromosomal abnormalities” by ultrasound examination during the fetal period, which does not show morphological abnormalities or multiple malformations that are often accompanied by chromosomal abnormalities, and no external surface malformations or clinical findings that are suspected to be chromosomal abnormalities after birth. Nonetheless, all cases with more than 12 weeks of gestation, and not just cases in the third trimester, were included in the study, ensuring that the cases of induced abortion due to prenatal diagnosis were also included. In this study, chromosomal tests were not performed in all cases, but at least chromosomal abnormalities that affect the phenotype could be extracted. In some cases, chromosomal abnormalities were not observed even if there were phenotypic abnormalities, such as multiple malformations.

In this study, DD and MD twins were compared based on membrane and not zygosity. Of note, 25–30% of monozygotic twins are DD twins [10]. Although DNA analyses of newborns are required to determine zygosity, chorionicity is more practical because it is determined by prenatal ultrasound. In clinical practice, the management of twin pregnancies depends on chorionicity. Therefore, the results of this study are clinically more useful.

This is the first attempt to investigate the frequency of chromosomal abnormalities in twin pregnancies in Japan. In most of the previous studies conducted in Europe and in the United States, only the incidence of Down syndrome was investigated [6, 21]. In contrast, in this study, the rate of all chromosomal aberrations in twin pregnancies was evaluated, which revealed that MD twins have a lower chromosomal aberration rate than DD twins. This study may provide useful information that could pave the way for better genetic counseling before prenatal testing.

## Data Availability

The data that support the findings of this study are available from the corresponding author upon reasonable request.
